# The interplay between personalities and social interactions affects the cohesion of the group and the speed of aggregation

**DOI:** 10.1371/journal.pone.0201053

**Published:** 2018-08-08

**Authors:** Isaac Planas-Sitjà, Stamatios C. Nicolis, Grégory Sempo, Jean-Louis Deneubourg

**Affiliations:** Biological and Artificial Self-organised Systems Team—CP 231, Université libre de Bruxelles (ULB), Campus Plaine, Boulevard du Triomphe, Bruxelles, Belgium; Arizona State University, UNITED STATES

## Abstract

Collective decision-making plays a central role in group-living animals and can be crucial to the survival of a group and the fitness of its members. As group-level properties emerge from individual decisions, personality variation can be a major determinant of collective behaviours. Here, we explore the relationship between personality and social interactions to explain the speed and cohesion of collective decision making during the aggregation process of the American cockroach (*Periplaneta americana*). We composed groups solely with shy individuals (spending a long time sheltered) or bold individuals (spending a short time sheltered) and tested them in a binary setup (arena with two shelters) for 3 consecutive days. We analysed the shelter use of individuals and groups to compare behavioural consistency among days and analyse the collective decision-making process. Contrary to the bold groups, shy groups had a faster aggregation process with more individuals sheltered mainly because shy individuals found the shelter more rapidly. Moreover, we show that personality is modulated by social interactions. We show high behavioural plasticity in bold groups, where some individuals act shy. This also suggests that learning and regulation mechanisms may take place. This study sheds some light on the implications of individual personality for collective decision making and the key role of shy individuals in gregarious species, such as *P*. *americana*.

## Introduction

Groups often decide collectively about vital activities such as foraging, migration [1–3], when or where to nest [4] and selecting a shelter in which to rest [5,6]. Indeed, the survival of the group and of the individuals composing it can depend on how these collective decisions are reached [7,8] and, in particular, how groups make optimal collective decisions based on limited information [8]. For instance, when choosing habitat in a patchy environment, group-living species are sometimes confronted with a choice between many sites offering the same habitat but differing in their intrinsic quality [9]. In such cases, social information can provide an accurate estimate of habitat quality [10,11]. Hence, public information (such as the presence of conspecifics) can provide a local social cue [12,13] that can be used by individuals to supplement their personal information [5,14–16].

Group-level properties (e.g., collective movements or decisions) emerge from individual decisions when individuals respond to their local environment and their neighbours [17–22]. Most studies focusing on this topic have not considered interindividual differences, potentially leading to misinterpretations as far as the mechanisms are concerned. The study of animal personality–the tendency for individuals to differ consistently in their behaviour through time and across contexts [23]–represents a major current topic in different fields, such as animal behaviour [24], behavioural ecology [25–28], and evolutionary biology [29–31]. Animal personality concerns a wide variety of traits [31] that are frequently linked to survival and longevity [32]. For instance, boldness refers to the extent to which individuals take risks when engaging in foraging, exploration or resource competition [33]. Variation in boldness may be maintained within populations as a result of strong growth-mortality trade-offs [34]. Boldness is frequently measured as the time spent vulnerable to predators (e.g., away from a refuge), with the boldest individuals being more exposed and thus believed to be at a greater risk. In addition, animal personality is often a major determinant of collective behaviours [35,36] and other group-level characteristics [37,38] in a wide variety of taxa, such as birds [39,40], fish [41,42] and invertebrates [43,44].

In this study, we use the American cockroach (*Periplaneta americana*) to investigate how the composition of personalities within a group affects the speed and the cohesion of collective decision making. Domiciliary cockroaches are a model species for the study of the aggregation process and decision making, and, more recently, personality variation [45–49]. Indeed, cockroaches obtain large benefits from this aggregation such as the dilution effect and a ‘many-eyes’ effect to detect predators or dangers, but they also benefit from a decrease in water loss, which is a major risk for this species [50,51]. A previous study showed individual personalities in *P*. *americana* during the aggregation process [48], with some individuals visiting shelters more often and aggregating faster than others. These consistent differences over time were observed for individuals within a group and for the groups themselves. Nevertheless, the relationship between individual personality and group-level properties, as well as the role of social interactions remained unclear, in particular whether the differences in exploration behaviour were due to individuals showing different light-sensitivity thresholds or to differences in activity rhythms.

We performed experiments with groups of cockroaches composed of individuals sharing the same personality (either all shy or all bold individuals) using the same setup as in a previous study [48] to shed light on the relationship between individual personality and group-level properties. We tested two hypotheses previously discussed in the literature to explain the emergence of consistent differences in collective behaviour [52,53]. The first one assumes individual personality variation within the group and social interactions (e.g., attraction to conspecifics) that are the same for all individuals. Different average individual behaviour would therefore lead to differences at the group level [48,54]. In such case, we predict that the groups composed with either all shy or all bold individuals will show different group-level properties according to their individual average behaviour. These differences will be mainly due to non-social behaviours like the probability of joining a shelter [5,48,54], the probability of leaving a shelter depending mainly on social interactions (e.g., retention effect). In this context, individual personality should then be maintained over days regardless of the composition of the group (e.g., shy or bold individuals). The second hypothesis assumes that individuals vary only in their social cohesion (e.g., more or less attracted to conspecifics) and that different social interaction networks within groups could generate differences in group behaviour [45]. Thus, in the case of groups composed by all shy or all bold individuals (having different social interaction network), we expect to observe important differences in their social behaviours such as the probability of leaving a shelter [5,46,55]. These differences could then be the consequence of shy individuals inducing a higher retention effect under shelters, through social interactions, than bold individuals.

## Methods

### Biological model

*Periplaneta americana* (L.) (Dictyoptera: Blattidae) is a nocturnal domiciliary cockroach that forms aggregates during daylight hours in dark and warm places. Adults measure 35–50 mm in length and even though they have wings, they rarely fly. The cockroaches used in this study were issued from strains reared in the breeding facilities of the Université libre de Bruxelles (ULB). These strains have been reared in the ULB since 2002 in five Plexiglas vivaria (80×40×100 cm) with cardboard tubes that hang from the walls to serve as shelters, and each vivarium contains about 1000 individuals of both sexes and of all developmental stages. The cockroaches were provided with dog pellets and water twice a week and the rearing room was maintained at 25±1°C under a 12:12 h light/dark cycle.

### Experimental setup

Experiments were carried out on adult males of *P*. *americana* without external damage. We used an experimental setup similar to the one used in [48]: a circular arena covered with a paper layer (120 g/m^2^), surrounded by a black polyethylene ring (diameter: 100 cm, height: 20 cm); the inner surface of this ring was covered by an electric fence to prevent cockroaches from escaping [55]. The lighting source (four Philips Ambiance Pro 20 W lamp bulbs) was placed above the arena and provided homogeneous illumination intensity. Two shelters made of transparent Plexiglas discs (diameter: 15 cm) were placed on the arena and covered by a red filter film (Rosco E-Colour 19:fire), creating low luminosity zones, perceived as rest sites for cockroaches, which are photophobic [50]. The centre of each disc was located 23 cm from the edge of the arena and 3 cm above the floor arena. Each shelter was large enough to potentially contain the entire group [55]. In order to detect when the insects were in the shelters, cockroaches were tagged with a RFID chip (diameter: 7.1± 0.2 mm and weight: 107± 3 mg; Spacecode) and a circular RFID reader was located below each shelter. The setup was surrounded by white curtains to avoid spatial cues (see S1 Fig and [48] for more details).

### Experimental procedure and measures

Groups of 16 males were kept in total darkness for 48 h in Plexiglas boxes (36 x 24 x 14 cm) containing a cardboard shelter, humidified cotton wool and *ad libitum* food. Afterwards, the cardboard shelter containing the 16 cockroaches was introduced to the centre of the arena (with lights already turned on) and opened to let cockroaches explore the arena. As we had two identical setups, two groups were tested at the same time for a first trial on Day 1. After this trial (each trial lasted 3h), we quantified the total time spent under the shelters for each cockroach (individual resting time or IRT). The 8 individuals of each group that spent the largest time sheltered, were put together to compose a new group of 16 cockroaches. Consequently, the remaining 8 individuals of each group that spent the shortest time under the shelters were put together to compose another new group. Thus, the two new groups were composed of shy individuals (long resting time) and bold individuals (short resting time). These newly composed groups were tested again on Day 3 and Day 5. During the 45 h gap between trials, the groups were kept in the dark in the same Plexiglas box. This procedure was repeated for 14 groups (7 bold groups and 7 shy groups). As a control condition, we did the same procedure with 8 groups without changing the composition of individuals within groups. Experiments were conducted during the two following periods: October 2013 –January 2014 and October 2014 –January 2015.

For Day 3 and Day 5 we measured the time spent under shelters for each individual (IRT). The group resting time (GRT) was the mean IRT for each group. Second, we counted the number of cockroaches present under each shelter every 10 minutes, allowing us to quantify the aggregation dynamics along the experiments. Finally, to identify the emergence of a consensus, we compared the distribution of cockroaches aggregated under each of two shelters at the end of experiments with a symmetrical binomial distribution. A consensus resulting from social interactions is reached when one of the two shelters contains a statistically higher number of sheltered individuals than expected under a symmetrical binomial distribution [48]. When there is no interaction between individuals, each individual should choose a shelter independently with probability 0.5, leading to a symmetric binomial distribution (i.e., no consensus).

### Analysis

We used Python 3.4.3 (Python Software Foundation, http://www.python.org) for data treatment and R software 3.2.2 (The R foundation for statistical computing, http://www.r-project.org/) for statistical analysis. At the individual level, we used a linear mixed model (LMM) to test the effects of condition and day for the sheltering time of individuals (IRT). The LMM with best AIC score was the one taking into account the interaction between condition and day (Condition * Day) and controlling for individuals and week as random effects ((1|individuals) + (1|week)). Finally, a linear model was used to generate the regression slopes between days and therefore assess behavioural consistency of the IRT. The F-test was used to test whether the slopes between Day 3 and Day 5 could be considered similar to the slopes between Day 1 and Day 3 for each condition. We used t-tests and Mann-Whitney tests to compare the change in number of sheltered cockroaches over time of the experiments. Finally, we used the binomial test to compare the fraction of cockroaches under each shelter at the end of the experiment to a theoretical random distribution (0.5 each shelter). A consensus was only reached if the observed fractions were significantly different from the theoretical one, meaning that individuals had selected a shelter for the majority. From these results we obtained the probability of reaching a consensus for each condition and we compared these probabilities between the shy, bold and control conditions with the Fisher Exact Probability test. The significance of statistical tests was fixed to α = 0.05.

## Results

**Fig 1:**
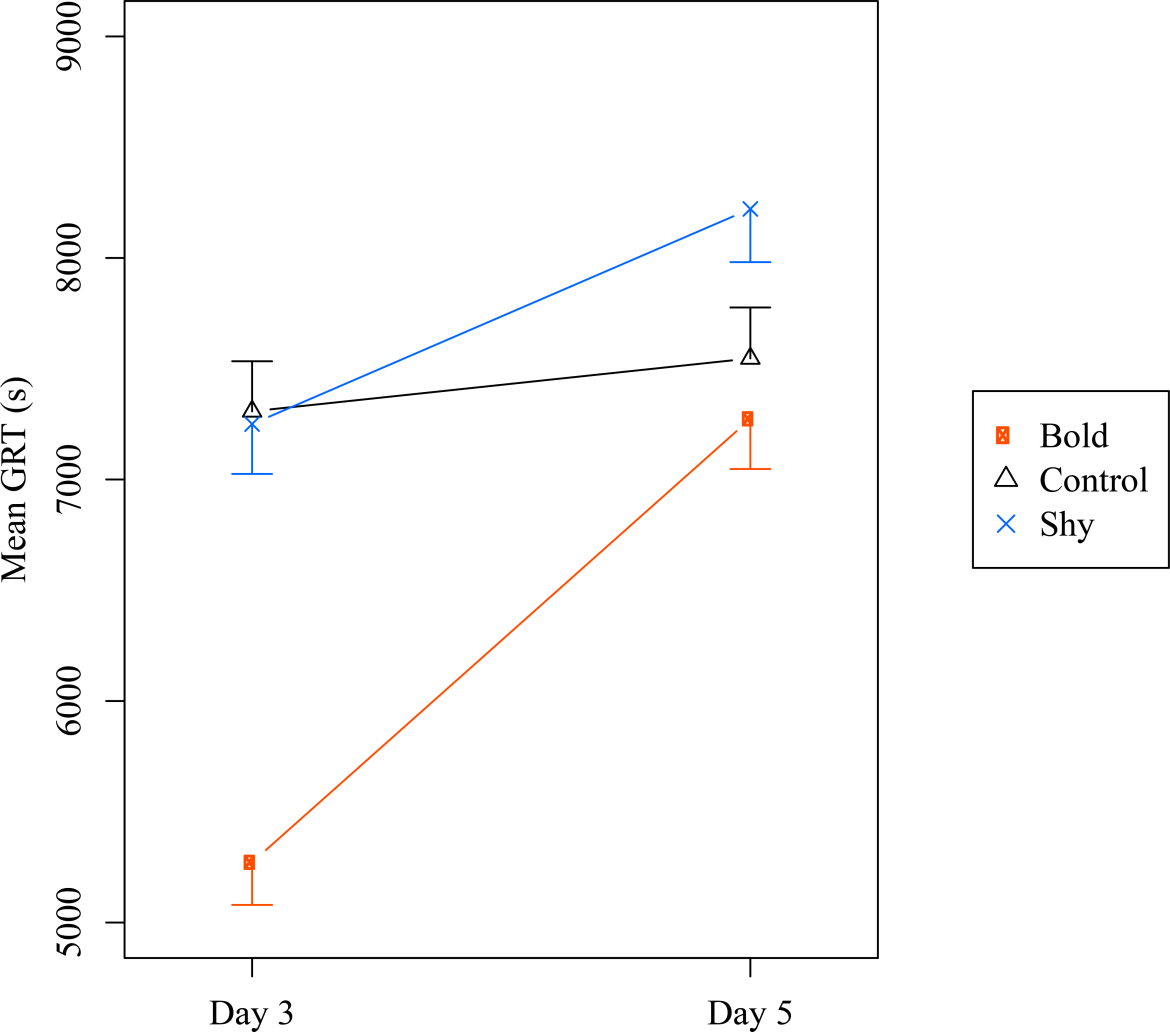
Mean GRT. Mean GRT (± SE) observed for the bold, control and shy conditions during Day 3 and Day 5. Lines indicate the increase in GRT between Day 3 and Day 5.

Once shy and bold groups were composed (see Methods section), we analysed the differences between the conditions (control, shy or bold) during Day 3 and Day 5. Day 1 was not included in the analysis as it was only used to select bold/shy individuals. The LMM shows that condition had a significant effect on the sheltering time of individuals (χ^2^ = 10.7, P = 0.005). The intercept for the bold condition was significantly less than that for the control condition (χ^2^ = -4.27, P <0.001) but not than that for the shy condition (χ^2^ = -0.12, P = 0.9). Thus, regarding Day 3, bold individuals spent less time sheltered than did control and shy individuals, which were not different from each other. Due to our methodology, every bold group was tested at the same time as one shy group, which allowed us to perform a visual pairwise comparison. Each shy group spent more time sheltered (greater GRT) than did the corresponding bold group tested during the same day (S2 and S3 Figs), which agrees with the results of the LMM. The Day factor also had an effect on the sheltering time of individuals (χ^2^ = 70.8, P<0.001), meaning that the IRT increased between Day 3 and Day 5 for at least one condition. The positive interaction between condition and day (χ^2^ = 45.36, P<0.001) shows that the shy and bold individuals increased their resting time at Day 5 with a greater increment for the bold individuals (Fig 1A). This effect was not observed for the control condition (χ^2^ = 1.353, P = 0.18; Fig 1).

**Fig 2:**
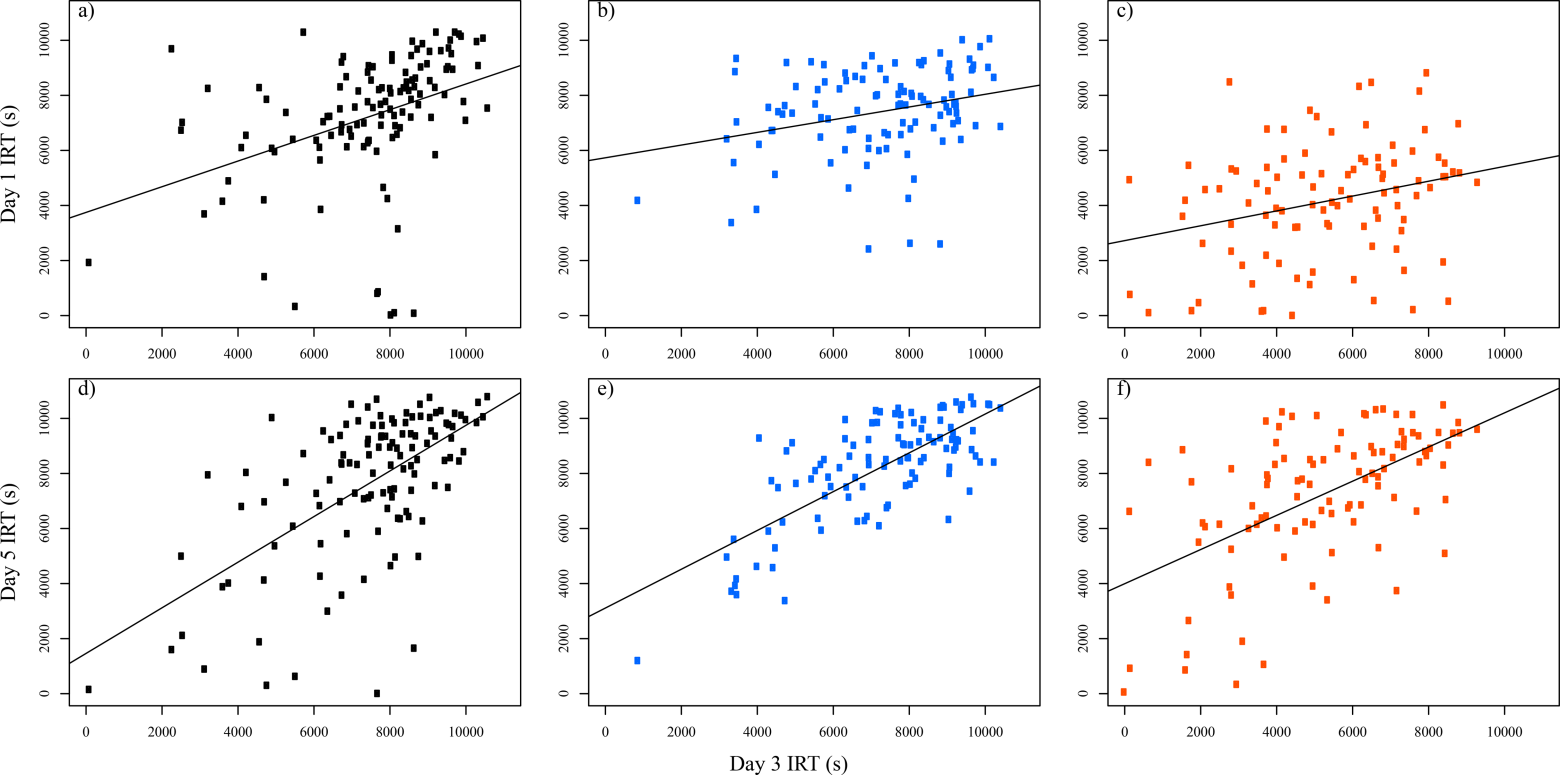
Correlation of the IRT between days. Correlation of the IRT between Day 1 and Day 3 for the a) control (3748 + 0.47x; R^2^ = 0.15), b) shy (5733+0.23x; R^2^ = 0.08) and c) bold (2723+0.28x; R^2^ = 0.08) conditions and between Day 3 and Day 5 for the d) control (1462+0.83x; R^2^ = 0.37), e) shy (3111+0.7x; R^2^ = 0.54) and f) bold (3995+0.62x; R^2^ = 0.32) conditions. The line shows the linear regression of the data.

One of our aims was to test the consistency of individual behaviours. We show that individuals have repeatable behaviour during experiments between successive days. Indeed, Fig 2 shows a positive correlation of the IRT between Day 1 and Day 3 (control: R^2^ = 0.15, P<0.001; shy: R^2^ = 0.08, P = 0.003; bold: R^2^ = 0.08, P = 0.005; see Fig 2A, 2B and 2C) and between Day 3 and Day 5 (control: R^2^ = 0.37, P<0.001; shy: R^2^ = 0.54, P<0.001; bold: R^2^ = 0.32, P<0.001; see Fig 2D, 2E and 2F). These results are in accordance with the results of the LMM, in which controlling for individuals with different intercepts significantly improved the model (χ^2^ = 98.1, P <0.001), and the repeatability of responses was *r* = 0.52. The correlation slope between Day 1 and Day 3 for individuals in the control groups cannot be considered different from the slope between Day 3 and Day 5 (F-test: F_1,236_ = 2.25, P = 0.1347; Fig 2A and 2D). Regarding the bold and shy conditions, the slope between Day 3 and Day 5 was significantly (or nearly significantly) greater compared to the slope between Day 1 and Day 3 (shy: F_1,208_ = 10.34, P = 0.002; bold: F_1,196_ = 3.28, P = 0.07; see Fig 2B, 2C, 2E, and 2F). Thus, the correlation was greater when the composition of the groups remained the same.

We also studied the effects of personality composition on the number of cockroaches in both shelters (global sheltering dynamics) and on the number of individuals settled in the selected shelter at the end of the experiment (cohesion). Eq 1 gives *P(t)*, the proportion of sheltered cockroaches at time *t*, and therefore describes the global sheltering dynamics, neglecting the social interactions, where μ corresponds to the maximum sheltered population and β is the growth rate of the sheltered population. The μ value is the ratio between the individual rate of joining the shelter and β (Eq 2), which is the sum of the joining rate (J) and the individual rate of leaving (L) (Eq 3). Thus, the higher the rate of joining the shelter, the higher the maximum population (μ). In contrast, the higher the rate of leaving, the lower the maximum population (μ). We used these equations to compare the sheltering and aggregation process between shy and bold groups.

$$P\left(t\right)=\mu \left(1-e^{-\beta \cdot t}\right)$$ (1)

$$\mu =\frac{J}{\beta }$$ (2)

β=J+L (3)

**Fig 3:**
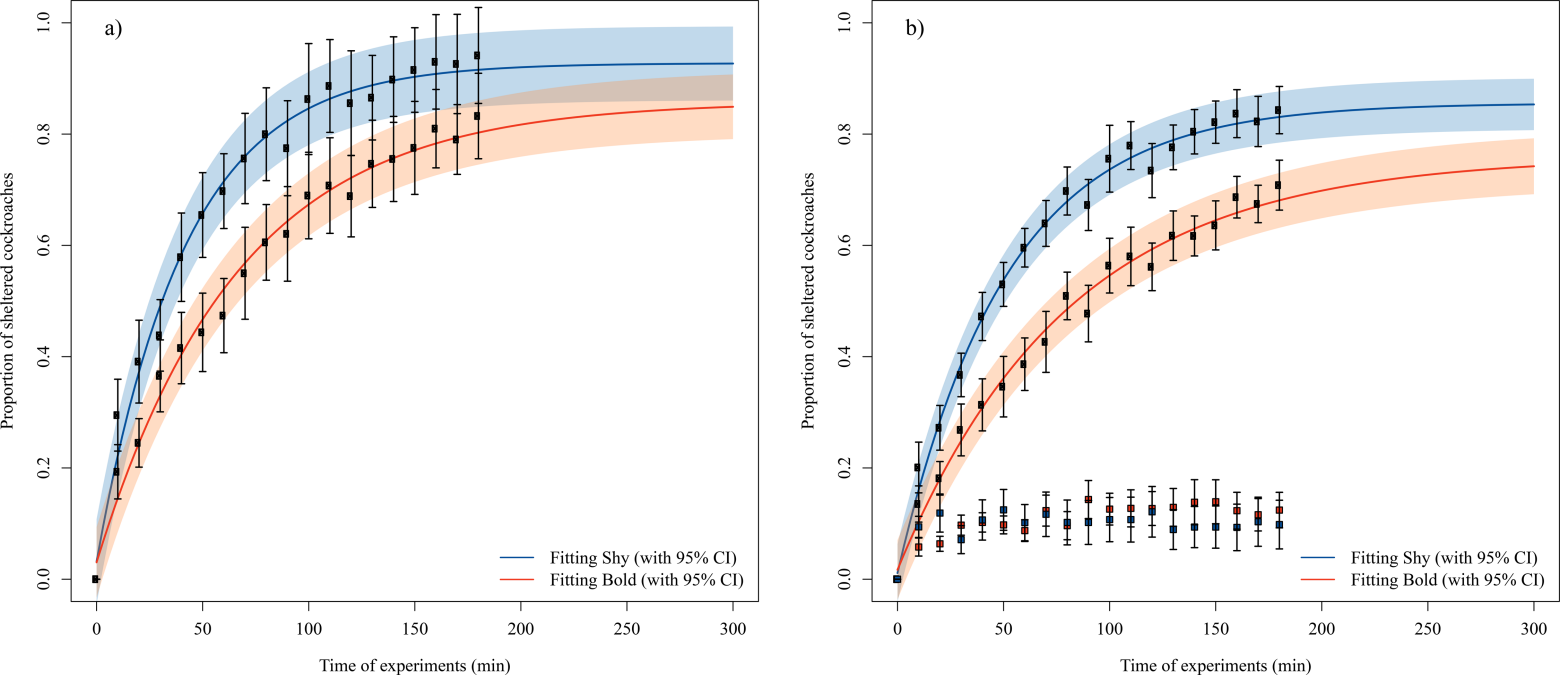
Change in the number of sheltered cockroaches over time in the experiments. We use the colour blue for the shy condition and orange for the bold condition. a) The mean proportion (± SE) of sheltered cockroaches every 10 minutes in both shelters for the shy and bold conditions. The theoretical proportions of total aggregated cockroaches (with 95% CI) obtained with Eq 1 was fitted for the shy (R^2^ = 0.83) and bold (R^2^ = 0.71) conditions. b) We represent in dots the mean proportion (± SE) of individuals in the selected shelter and in squares the proportion of cockroaches in the unselected shelter. The theoretical proportions of aggregated cockroaches (with 95% CI) in the selected shelter (Eq 1) were fitted for the shy (R^2^ = 0.7) and bold (R^2^ = 0.59) conditions.

We fitted Eq 1 to the global sheltering behaviour data (Fig 3A) to estimate μ and β values for groups of shy and bold cockroaches. This fitting shows that the μ and β values (shy: μ = 0.93±0.014, β = 0.024±0.0013, R^2^ = 0.83; bold: μ = 0.86±0.03, β = 0.015±0.0014, R^2^ = 0.71) were significantly larger for the shy condition than for the bold condition (t-test for μ: t_10_ = 4.58, P = 0.005; t-test for β: t_10_ = 10.24, P<0.001). In agreement with these results, the final proportion of cockroaches sheltered under both shelters was larger for the shy groups than for the bold groups (Mann-Whitney test: U = 151, P = 0.015). The μ and β values obtained allowed us to compute the joining (J) and leaving (L) rates. Interestingly, we see that the individual joining rate (Eq 2; J_shy_ = 0.023, J_bold_ = 0.013) is larger and the leaving rate (Eq 2; L_shy_ = 0.0017, L_bold_ = 0.0022) is lower for the shy condition, suggesting that shy individuals joined the shelter faster and remained longer under the shelters.

We used the same equations (Eqs 1, 2 and 3) to analyse the sheltering behaviour under the selected shelter. The aggregation dynamics of shy groups were the fastest (β_shy_ = 0.02±0.0017, β_bold_ = 0.012±0.0018; t-test: t_10_ = 3.68, P = 0.01) and had the highest plateau value (μ_shy_ = 0.86±0.025, μ_bold_ = 0.77±0.05, t-test: t_10_ = 6.54, P<0.001). Not surprisingly, the proportion of individuals aggregated under the selected shelter was larger for the shy groups than for the bold groups (Mann-Whitney test: U = 48, P = 0.023; Fig 3). Moreover, the rate of joining was larger for the shy individuals than the bold individuals (J_shy_ = 0.017; J_bold_ = 0.01), but the rate of leaving was similar for both conditions (L_shy_ = 0.0028; L_bold_ = 0.0029), suggesting that the shy individuals joined the shelter faster but did not remain longer, as expected from the general dynamics. This indicates that the differences in the probability of leaving the shelter observed in the general dynamics are due to the cockroaches visiting the unselected shelter. The dynamics of aggregation under the unselected shelter were not analysed due to the low number of cockroaches that aggregated (mean of 1–2 individuals), which gave unreliable fittings. Nevertheless, we were able to compare the final proportions of cockroaches that settled in this shelter, and they were not different between the conditions (Mann-Whitney test: U = 119, P = 0.33; Fig 3).

We considered the consensus to be reached only when the fraction of the population that settled under the selected shelter was significantly higher than 0.5. The proportion of groups that reached a consensus at the end of experiment was not different between the shy (0.75), bold (0.75) and control (0.67) conditions (F-test: df = 2, P = 0.81; see S1 Table).

## Discussion

In this study, we tested two hypotheses explaining how personality is maintained within groups leading to differences at group level: (1) variation in average individual behaviour and (2) variation in social interaction networks within groups. We showed that the composition of the group (shy or bold individuals) affected the sheltering time of the whole group as well as the aggregation dynamics of the groups (Figs 1 and 3; see also S2 Fig). Moreover, shy and bold individuals showed different joining rate, which is not affected by social interactions as individuals cannot perceive the quality of the shelter before joining it [see also 5]. These results support the first hypothesis that differences in mean individual behaviour induce differences at the group level [52] and agrees with previous studies showing individual personalities in *P*. *americana* [48,49,54]. The second hypothesis predicts that groups composed by all shy or all bold individuals differ in their cohesion and their network of social interactions, which should in turn affect the individual and group behaviour. The aggregation process in cockroaches is mainly characterised by inter-attraction between individuals [56,57] through the hydrocarbons found on their body surface (e.g., [58]), the quantity of which may vary between individuals. If shy and bold individuals promoted different levels of attraction and/or response to conspecifics, the strength of social interactions could depend on group composition and affect aggregation dynamics. In such case, shy and bold individuals should show different leaving rates, as it depends on the retention effect of congeners under the shelter. Our results show that the leaving rate of shy and bold individuals under the selected shelter (where aggregation takes place) is comparable, suggesting that shy and bold individuals were similarly retained by other individuals and that social interactions cannot be considered different. On the other hand, if social interactions were identical for both phenotypes, individual behaviour should be equally repeatable between Day 1 and Day 3 (composition of the groups changed) than between Day 3 and Day 5. Instead, we observed a lower stability of personality variation between Day 1 and Day 3 than between Day 3 and Day 5 in the shy and bold conditions (Fig 2). This effect was not observed in the control condition, in which the composition of the groups remained unchanged over the week. In other words, modifying the group composition between Day 1 and Day 3 may explain the weak consistency between these two days in the shy and bold conditions. These results, in agreement with previous studies in other species [45,59], suggest that even if social interactions cannot be considered different between individuals, they play a role on individual personality and group-level properties.

For analysing global sheltering dynamics, our procedure based on relative sorting (characterising 50% of individuals as bold and 50% as shy) was able to establish different aggregation dynamics. The shy individuals settled faster under shelters (larger joining rate) and had longer stays (smaller leaving rate) than did the bold individuals. These results are in agreement with the hypothesis that shy individuals try to reduce light exposure as they are more sensitive to it. In addition, these results are in contradiction with an alternative hypothesis assuming that shy individuals are more active and therefore that these individuals have a larger joining rate as well as a larger leaving rate than bold individuals. A fine-grained analysis of the leaving rate from the selected shelter, where most of social interactions are at work, shows that these differences tend to disappear. Further physiological studies could give more information about the proximal mechanisms taking place and generating differences in individual sheltering dynamics. Our results suggest that shy individuals retain the bolder ones by spending more time sheltered under shelters and therefore strengthen the social facilitation of individuals [60]. Shy individuals, by sheltering faster and spending more time sheltered, will promote more attraction to a shelter than will bolder individuals, which spend less time sheltered; shy individuals will therefore act as keystone individuals [61] with a disproportionally large effect on other group members and group dynamics.

The shy and bold groups had the same probability of reaching consensus, in agreement with a previous study [48]. Interestingly, shy groups were more cohesive when reaching this consensus, with faster aggregation and with a larger population in the selected shelter compared to bold groups (Fig 3). The fitting of Eq 1 shows that these differences are mainly due to differences between joining probabilities, which is also in agreement with previous studies [48,49,54]. Even when using two equal options (i.e., identical shelters), differences in cohesion and in the way consensus is reached may have an important impact on individual survival. Indeed, due to the cooperative behaviour of gregarious species, individuals produce a small difference in occupation between shelters that is later amplified. Higher occupation confers benefits such as diminished water loss and dilution of predation risk. Fast aggregation of a larger number of cockroaches may enhance these benefits [50,62].

The trade-off between speed and accuracy has been studied extensively in the context of different-quality options and often neglects animal personality [4,63,64]. Based on the literature [5,48,65] and our results, one natural prediction is that in an experimental setup where groups would have to choose between two different quality shelters (i.e., each one giving a different trade-off), a shy group would rapidly reach a consensus but with low accuracy (lower frequency of selection of the best shelter). Taking into account the costs and benefits resulting from the speed-accuracy trade-off, these experiments would improve the understanding of the evolution and selection of behavioural types [66].

Regarding individual behaviour, we observed an increase in the sheltering time across days for the bold and shy groups. Interestingly, this increment was larger for the bold individuals than for the shy ones. This interaction between days and conditions (see the LMM results) is an interesting explanation for the role of shy individuals within a group and for how different behavioural phenotypes affect interactions between them [60]. We hypothesise that regulation mechanisms are taking place in bold groups: a fraction of the population starts acting shy in order to favour aggregation. Indeed, the distribution of the IRT within the bold groups at Day 5 was similar to the ones within the control groups (i.e., Day 1, 3 and 5), suggesting that some individuals originally identified as bold could act shy in the newly composed bold group. The existence of this regulation is supported by the fact that behavioural consistency was higher when the composition of the group remained the same. On the other hand, this potential regulation seemed to be absent for the shy and control groups, which increased their sheltering time by a minor degree (Fig 1; see also S2 and S3 Figs). This suggests that other mechanisms may take place, such as the recognition of and habituation to the setup. Indeed, spatial orientation and olfactory learning abilities have been shown in domiciliary cockroaches [67,68]. These learning processes may be at work in our experiments and may occur under all conditions.

Personality variation within a population has been suggested to affect a wide range of ecological and evolutionary processes (e.g., population growth and stability, species interactions, community dynamics and social evolution) [28,36,69]. In this work, we highlight the importance of exploration behaviour and sheltering time in characterising and predicting personalities within a group in the case of domiciliary cockroaches. We also show that individual differences in reaction to (a) the environment and (b) conspecifics explain the relationship between personality variation and collective behaviour. Our conclusion is that the interplay between personality and social behaviour is crucial to explain the aggregation dynamics in domiciliary cockroaches, shedding light on the ecological and evolutionary success of gregarious insects [70].

## Supporting information

S1 Fig Experimental setup. Design of the experimental setup in a perspective and lateral view. A: Arena of the setup; S: Shelters with red filter; R: RFID reader. Setup identical to the one used in Planas-Sitjà et al. (2015) Proc Roy Soc B.
(PDF)

S2 Fig Group Resting Time (GRT) for each experimental group. Shy (blue) and bold (orange) groups are paired regarding the week they were tested.
(PDF)

S3 Fig IRT Boxplots. Boxplots showing the individual resting time (y-axis) of the individuals within group (x-axis, 7 groups) of the bold and shy conditions (cond) for A) Day 3 and B) Day 5.
(PDF)

S1 Table Binomial test results. Table with the Binomial test results for control, shy and bold conditions. Significance of P-value is indicated as (*) P<0.05; (**) P<0.01; (***) P<0.001.
(PDF)
