# Supplementary material for: The interplay between personalities and social interactions affects the cohesion of the group and the speed of aggregation
Source: PLoS One. 2018 Aug 8;13(8):e0201053. doi: 10.1371/journal.pone.0201053 (PMC6082527; doi:10.1371/journal.pone.0201053)
Supplement: S1 Fig — Design of the experimental setup in a perspective and lateral view. A: Arena of the setup; S: Shelters with red filter; R: RFID reader. Setup identical to the one used in Planas-Sitjà et al. (2015) Proc Roy Soc B. (PDF) [file pone.0201053.s001.pdf]

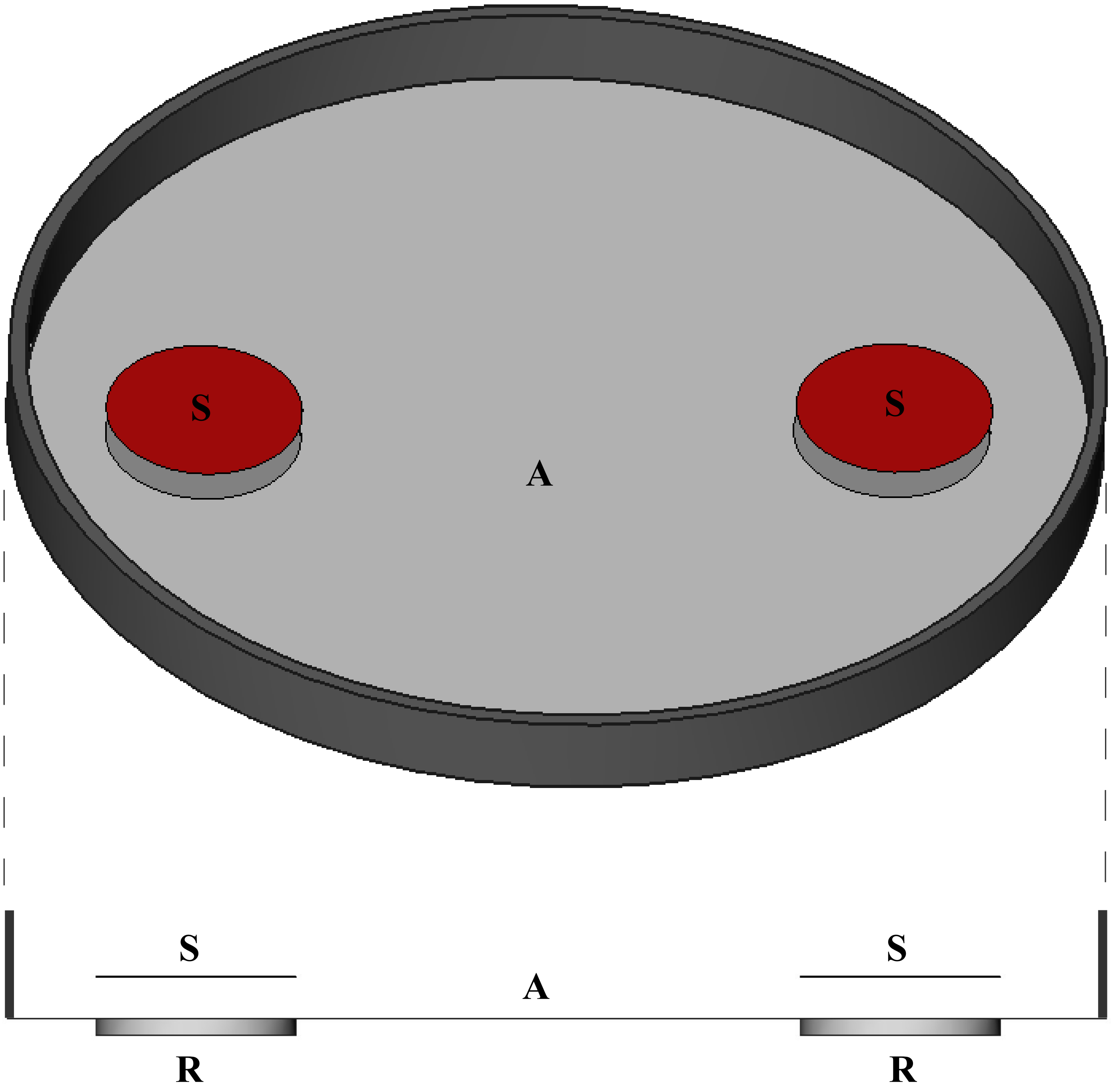

S1 Fig. Experimental Setup. Design of the experimental setup in a perspective and lateral view. A: Arena of the setup; S: Shelters with red filter; R: RFID Reader. Setup identical to the one used in Planas-Sitjà et al. (2015) Proc Roy Soc B.
