# Supplementary material for: The interplay between personalities and social interactions affects the cohesion of the group and the speed of aggregation
Source: PLoS One. 2018 Aug 8;13(8):e0201053. doi: 10.1371/journal.pone.0201053 (PMC6082527; doi:10.1371/journal.pone.0201053)
Supplement: S2 Fig — Shy (blue) and bold (orange) groups are paired regarding the week they were tested. (PDF) [file pone.0201053.s002.pdf]

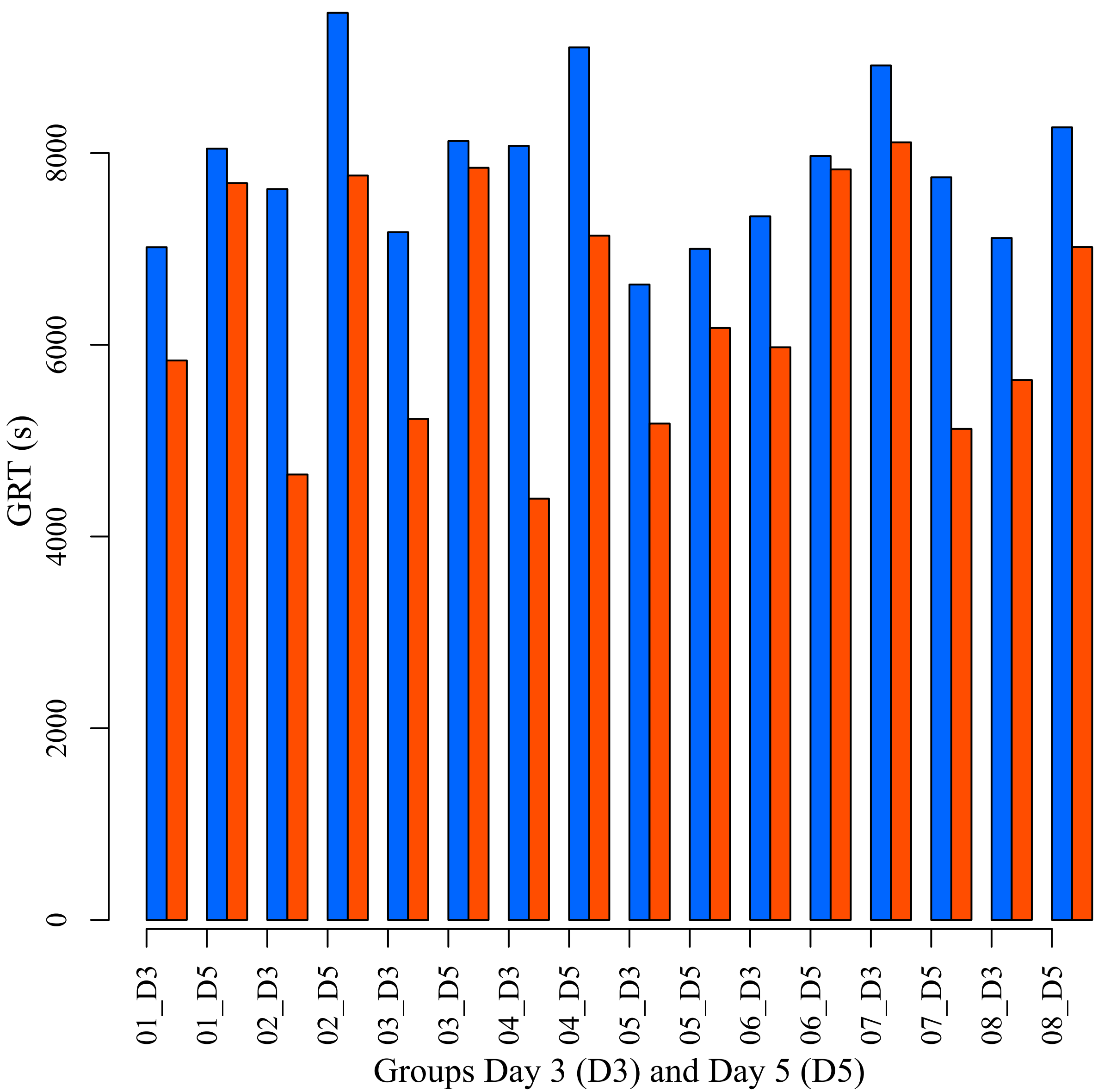

S2 Fig. Group Resting Time (GRT) for Each Experimental Group. Shy (blue) and bold (orange) groups are paired regarding the week they were tested.
