# Supplementary material for: The interplay between personalities and social interactions affects the cohesion of the group and the speed of aggregation
Source: PLoS One. 2018 Aug 8;13(8):e0201053. doi: 10.1371/journal.pone.0201053 (PMC6082527; doi:10.1371/journal.pone.0201053)
Supplement: S3 Fig — Boxplots showing the individual resting time (y-axis) of the individuals within group (x-axis, 7 groups) of the bold and shy conditions (cond) for A) Day 3 and B) Day 5. (PDF) [file pone.0201053.s003.pdf]

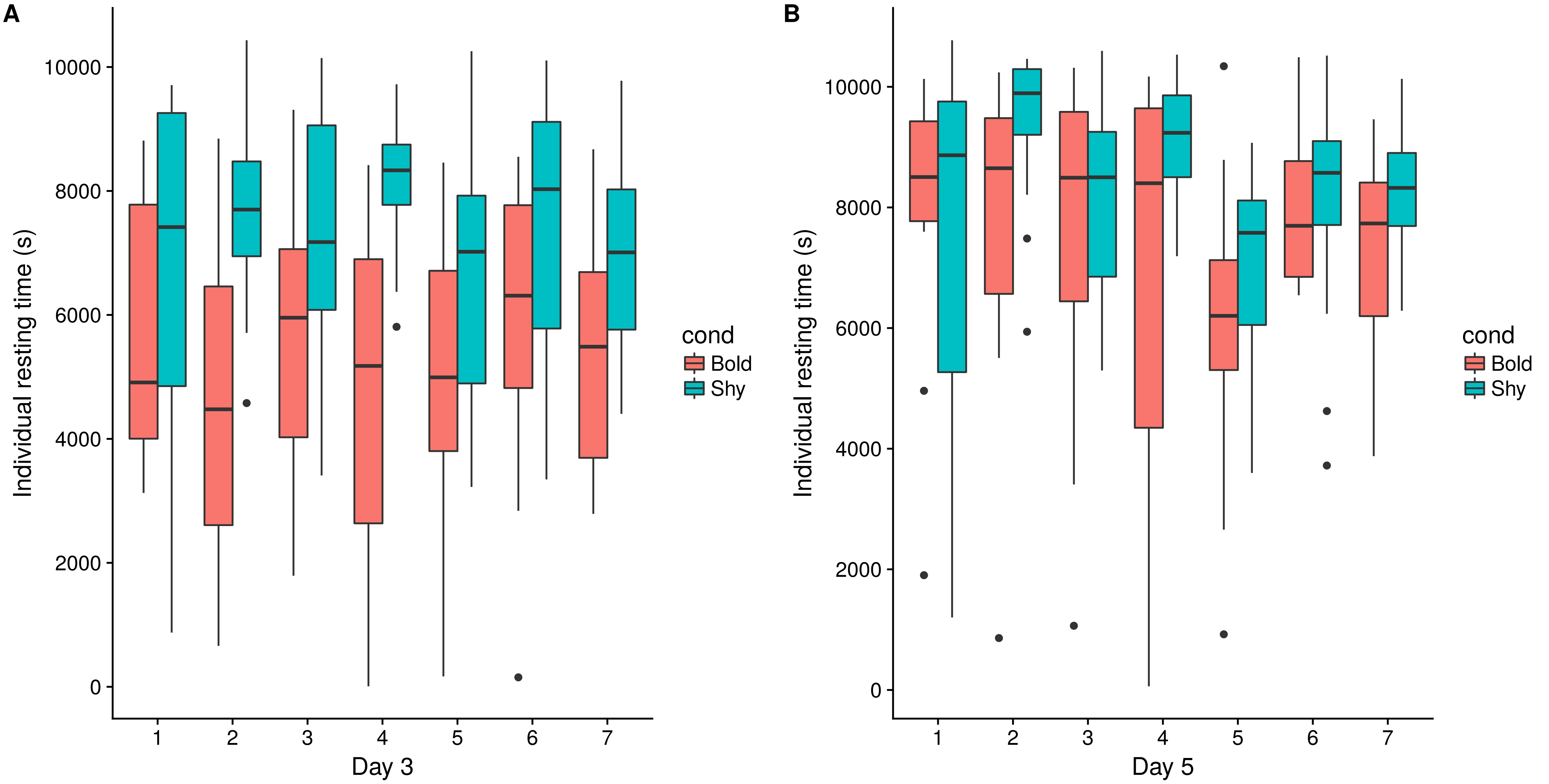

S3 Fig. IRT Boxplots. Boxplots showing the individual resting time (y-axis) of the individuals within groups (x-axis, 7 groups) of the bold and shy conditions (cond) for A) Day 3 and B) Day 5.
