# Supplementary material for: The interplay between personalities and social interactions affects the cohesion of the group and the speed of aggregation
Source: PLoS One. 2018 Aug 8;13(8):e0201053. doi: 10.1371/journal.pone.0201053 (PMC6082527; doi:10.1371/journal.pone.0201053)
Supplement: S1 Table — Table with the Binomial test results for control, shy and bold conditions. Significance of P-value is indicated as (*) P<0.05; (**) P<0.01; (***) P<0.001. (PDF) [file pone.0201053.s004.pdf]

S1 Table. Binomial Test Results.

Control Condition				Shy Condition				Bold Condition			
Shelter 1	Shelter 2	P-value	Signif.	Shelter 1	Shelter 2	P-value	Signif.	Shelter 1	Shelter 2	P-value	Signif.
6	9	0.153		11	0	<0.001	***	5	7	0.193	
14	1	<0.001	***	5	9	0.122		2	8	0.044	*
12	3	0.014	*	9	7	0.175		0	11	<0.001	***
9	8	0.185		10	6	0.122		8	4	0.121	
13	3	0.009	**	12	2	0.006	**	10	2	0.016	*
12	4	0.028	*	0	14	<0.001	***	0	12	<0.001	***
10	4	0.061		0	15	<0.001	***	0	11	<0.001	***
2	10	0.016	*	0	15	<0.001	***	0	11	<0.001	***
13	0	<0.001	***	0	13	<0.001	***	3	11	0.022	*
5	10	0.092		0	15	<0.001	***	6	9	0.153	
6	9	0.153		0	14	<0.001	***	2	13	0.003	**
3	12	0.014	*	0	13	<0.001	***	1	15	<0.001	***
10	4	0.061		7	7	0.209		14	0	<0.001	***
7	8	0.196		0	10	<0.001	***	0	3	0.125	
2	10	0.016	*	14	1	<0.001	***	0	9	0.002	**
0	9	0.002	**	14	0	<0.001	***	1	11	0.003	**
7	9	0.175									
1	13	<0.001	***								
0	12	<0.001	***								
13	1	<0.001	***								
11	3	0.022	*								
11	0	<0.001	***								
15	0	<0.001	***								
14	1	<0.001	***								

Table with the Binomial test results for control, shy and bold conditions.

Significance of P-value is indicated as (*) P<0.05; (**) P<0.01; (***) P<0.001
